# Diagnosis of Diabetes Mellitus Using Gradient Boosting Machine (LightGBM)

**DOI:** 10.3390/diagnostics11091714

**Published:** 2021-09-19

**Authors:** Derara Duba Rufo, Taye Girma Debelee, Achim Ibenthal, Worku Gachena Negera

**Affiliations:** 1 College of Engineering and Technology, Dilla University, Dilla 419, Ethiopia; derara2015duba@gmail.com; 2 College of Electrical and Mechanical Engineering, Addis Ababa Science and Technology University, Addis Ababa 120611, Ethiopia; tayegirma@gmail.com; 3 Ethiopian Artificial Intelligence Center, Addis Ababa 40782, Ethiopia; worku.gachena2@gmail.com; 4 Faculty of Engineering and Health, HAWK Universityof Applied Sciences and Arts, 37085 Göttingen, Germany

**Keywords:** diabetes mellitus, detection, LightGBM, diabetes diagnosis

## Abstract

Diabetes mellitus (DM) is a severe chronic disease that affects human health and has a high prevalence worldwide. Research has shown that half of the diabetic people throughout the world are unaware that they have DM and its complications are increasing, which presents new research challenges and opportunities. In this paper, we propose a preemptive diagnosis method for diabetes mellitus (DM) to assist or complement the early recognition of the disease in countries with low medical expert densities. Diabetes data are collected from the Zewditu Memorial Hospital (ZMHDD) in Addis Ababa, Ethiopia. Light Gradient Boosting Machine (LightGBM) is one of the most recent successful research findings for the gradient boosting framework that uses tree-based learning algorithms. It has low computational complexity and, therefore, is suited for applications in limited capacity regions such as Ethiopia. Thus, in this study, we apply the principle of LightGBM to develop an accurate model for the diagnosis of diabetes. The experimental results show that the prepared diabetes dataset is informative to predict the condition of diabetes mellitus. With accuracy, AUC, sensitivity, and specificity of 98.1%, 98.1%, 99.9%, and 96.3%, respectively, the LightGBM model outperformed KNN, SVM, NB, Bagging, RF, and XGBoost in the case of the ZMHDD dataset.

## 1. Introduction

Type 2 diabetes is the most common type of diabetes. As per the report by the International Diabetes Federation (IDF) 9th edition, there are currently 351.7 million people of working age (20–64 years) with diagnosed or undiagnosed diabetes in 2019, of which about 90% are type 2 diabetes. The number of people affected by diabetes is expected to increase to 417.3 million by 2030 and 486.1 million by 2045. The largest increase will take place in low and middle-income countries. Consequently, DM has become a life-threatening global health issue, which requires early detection and diagnosis to better prevent and reduce its incidence.

In the last few decades, many advanced data mining algorithms and data analysis techniques have been developed in the medical field, among others. Data mining technology has become an essential tool in the medical field for applications such as disease prediction, assistant diagnosis, breast cancer detection, brain tumor detection, and treatment [1,2,3,4,5,6,7,8,9,10]. Data mining technology extracts the knowledge and patterns hidden by diseases by analyzing a large amount of medical diagnostic data. Thus, it provides accurate decision-making for the diagnosis and handling of diseases. As the scale and complexity of medical data increases, the detection of diabetes mellitus using data mining becomes an important and interesting research problem.

In 2017, about 318,000 mobile health applications were available to consumers throughout the world [11]. This includes tools enabling diabetes self-management by mobile devices such as mobile phones, tablets, or smart watches [12]. Some diabetes applications differ in the choice of indicators to be tracked, such as blood glucose estimations, sustenance and sugar, physical movement and weight tracking, imparting information to health and social workers, as well as providing patient information. However, most of these existing diabetes-related mobile health applications are designed for users with a preceding affirmative diagnosis of the disease status and accompanying factors, while this study is dedicated to the early diagnosis of DM using machine learning algorithms.

There are several machine-learning-based diabetes assessment approaches; among them, diagnosis, prediction, and complication analysis are the most researched ones. In diabetes diagnosis [13,14,15], researchers used a patient’s diabetes history and physical examination results such as plasma glucose concentration, diastolic blood pressure, body mass index, age, weight, diet, insulin, water consumption, blood pressure, sex, etc. as input to the machine learning algorithms. The most frequently used machine learning algorithms are support vector machines (SVM), k-nearest neighbor (kNN), decision trees, Naive Bayes (NB), and tree boosting algorithms such as XGBoost, Adaboost, and random forest (RF) [15]. Conventional algorithms such as kNN, SVM, NB, etc. result in low performance, whereas ensemble algorithms such as XGBoost, Adaboost, and RF comparatively achieve a higher level of accuracy. Since these ensemble learners are defined on a set of hyperparameters, their design involves a global optimization task to combine a set of indicators into a reliable classification model.

Ravaut et al. [16] performed large-scale machine learning studies with health record datasets of patients in Ontario, Canada, provided by the Institute of Clinical Evaluative Sciences (ICES) to predict the risk of diabetes in a range of 1–10 years ahead. The considered dataset has about 963 total input features. The authors compared logistic regression, XGBoost, Highway Network, CNN-LSTM, and LSTM-Seq2Seq algorithms to predict the risk of diabetes mellitus for a scope of 10 years. Based on experimental analysis, the XGBoost model outperforms other algorithms. The most researched diabetes complications are retinopathy, neuropathy, and nephropathy. In [17], logistic regression is used to predict the involvement of retinopathy, nephropathy, and neuropathy at different time scenarios—3, 5, and 7 years from first diabetes reference. Input features are gender, hypertension, age, glycated hemoglobin (HbA1c), smoking habit, time from diagnosis (how long after diabetes diagnosis), and body mass index (BMI).

As discussed above, ensemble learning algorithms in many cases outperform other machine learning approaches for disease diagnosis. Fundamentally, this is achieved by combining multiple base classifiers (individual classifier algorithms) into an ensemble model by learning the inherent statistics of the combined classifiers and, hence, outperforming the single classifiers [18]. In this paper, we investigate LightGBM ensemble classifiers for the early detection of DM. This research work aims at supporting health practitioners in the diagnosis of DM.

LightGBM is an ensemble algorithm developed by Microsoft that provides an efficient implementation of the gradient boosting algorithm [19]. The primary benefit of LightGBM is a dramatic acceleration of the training algorithm, which, in many cases, results in a more effective model. LightGBM is constructed on the top of decision tree algorithms, employing $n_{estimators}$ numbers of boosted trees. Tree boosting algorithms outperform others for prediction problems [20]. The LightGBM ensemble learning algorithm has been applied in numerous classification and regression studies and achieved excellent detection results, indicating that LightGBM is an effective classifier algorithm.

The proposed LightGBM model provides an optimized decision-support system for users. The particularity of the proposed approach is in the procedure used to calculate the number of decision trees, maximum depth of the trees, and number of tree leaves to construct an optimal LightGBM model. Furthermore, the first local diabetes dataset of Ethiopia has been prepared to design a CAD (Computer Aided Diagnosis) system for the early detection of DM. Thus, the purpose of this study is to develop an optimal and accurate diabetes diagnosis model based on machine learning algorithms.

The remainder of this article is organized as follows: Section 2 discusses the related existing work and accomplishments in the prediction and diagnosis of DM. Section 3 describes the materials used in the experiment, the research method, and the details of the proposed diabetes detection model. Section 4 provides a discourse to the experimental results and model evaluation, including a comparison to previous research approaches. Section 5 states the study limitations and concludes the study with established guidelines for future work.

## 2. Related Work

In general, we found that there are two categories of existing methods related to diabetes prediction problems: machine learning viz. classification/detection [18,21,22,23] and forecasting or forward prediction [16]. In this study we are interested in estimating the probability of diabetes positivity and to review relevant indicators and machine learning methods.

From the existing publications, we generalized two main approaches related to diabetes-related features. In the first approach, some indicators that were more relevant to diabetes mellitus from the view of medicine are selected manually/systematically and used for diabetes prediction or diagnosis [21,22,23,24]. In the second approach, all diabetes-related available attributes are given to machine (deep) learning algorithms [16,25,26] and learning models must recognize the important features [16]. Our investigations follow the first approach by obtaining the expertise of physicians on diabetes indicators for data collection. The proposed indicators are verified by their correlation to the class variable in Table 1 in order to prove statistical relevance.

According to this survey, Deep Neural Networks (DNN) and Support Vector Machines (SVM) achieve the best classification outcomes, followed by random forests and other ensemble classifiers. For DM detection/prediction, the best-in-class method reported by Chaki et al. applies SVM on oral glucose tolerance test data at an accuracy of 96.8% [27]. Hence, this is regarded as a performance landmark for our algorithmic studies based on patient anamnesis data used to predict type 2 DM. Subsequently, we refer to studies on comparable data.

Deberneh and Kim [28] investigated the problem if patients will develop type 2 DM one year after data elicitation of 12 features: (*i*.) fasting plasma glucose, (*ii*.) glycated hemoglobin (HbA1c), (*iii*.) triglycerides, (*iv*.) body mass index, (*v*.) gamma-glutamyltranspeptidase (γ-GTP), (*vi*.) age, (*vii*.) uric acid, (*viii*.) sex, (*ix*.) smoking, (*x*.) drinking, (*xi*.) physical activity, and (*xii*.) family history. They found that the prediction has an accuracy of up to 73% for soft voting and random-forest-based approaches, while XGBoost performed slightly less at 72% accuracy. In case the input data are elicited over a period of the past 4 years, the accuracy increased to 81%. On the one hand, this is significantly less than the 96.8% prediction accuracy reported in [27]; on the other hand, the merits are to predict the occurrence of type 2 DM in the future and, hence, to allow for preventive treatment.

Chaki, J. et al. [29] systematically reviewed the art of machine learning and artificial intelligence for diabetes mellitus detection and self-management. Their work focused on four specific aspects: (*i*.) databases, (*ii*.) ML-based classification and diagnostic methods, (*iii*.) AI-based intelligent assistants for patients with DM, and (*iv*.) performance metrics.

Alasaf et al. [30] proposed a system aimed at preemptively diagnosing DM in Saudi Arabia. They retrieved data from King Fahd University Hospital (KFUH) in Khobar, Saudi Arabia. The collected dataset contained 399 records, of which 191 instances were diabetic and 208 instances were not diabetic with a binary target variable (diabetic or not). Preprocessing techniques were applied to the data to identify relevant features, and 48 more relevant features were selected and prepared for the identification/classification process. Four classification algorithms (SVM (LibSVM), ANN, NaiveBayes, and k-NN) were applied to predict the DM. As a result, ANN outperformed other algorithms with the testing accuracy of 77.5%.

Faruque et al. [31] explored various risk factors related to diabetes mellitus using machine learning techniques. They collected diabetes data of 200 patients consisting of 15 diabetes indicators (features): (*i*.) age, (*ii*.) sex, (*iii*.) weight, (*iv*.) diet, (*v*.) polyuria, (*vi*.) water consumption, (*vii*.) excessive thirst, (*viii*.) blood pressure, (*ix*.) hypertension, (*x*.) tiredness, (*xi*.) vision problems, (*xii*.) kidney problems, (*xiii*.) hearing loss, (*xiv*.) itchy skin, and (*xv*.) genetics with one binary class variable (diabetic or not) from the diagnostic of Medical Centre Chittagong, Bangladesh. Four machine learning algorithms (SVM, NB, KNN, and C4.5 Decision Tree) were used to predict diabetic mellitus. Empirical results showed that the C4.5 decision tree achieved a higher accuracy of 73.5% compared with other machine learning techniques.

Xu and Wang [18] proposed a type 2 diabetes risk prediction model based on an ensemble learning method using the publicly available UCI Pima Indian diabetes dataset (PIDD). PIDD contains eight diabetes indicator attributes viz. (*i*.) number of times pregnant, (*ii*.) plasma glucose concentration a 2 h in an oral glucose tolerance test, (*iii*.) diastolic blood pressure (mmHg), (*iv*.) triceps skin fold thickness (mm), (*v*.) 2-h serum insulin [μ U/mL] [32] (*vi*.) body mass index (weight (kg)/(height (m))${}^{2}$), (*vii*.) diabetes pedigree function, and (*viii*.) age (years) with one binary class variable (diabetic or not). They followed a two-step approach. Firstly, they developed a weighted feature selection algorithm based on random forest (RF-WFS) for optimal feature selection; then, the extreme gradient boosting (XGBoost) classifier was applied to predict the risk of diabetes mellitus accurately. The experimental results showed that the model has a better accuracy of 93.75% in classification performance than other preceding research results.

Nowadays, for classification and diagnosis problems, LightGBM outperforms other state-of-the-art methods, cf. [33,34,35,36,37,38,39,40]. In these related works, LightGBM is not only selected for its effective prediction performance, but also for its shorter computational time and optimized data handling technique. For instance, in [41], LightGBM and XGBoost algorithms were employed to construct the prediction models for cardiovascular and cerebrovascular diseases prediction based on different indicator elements (features) such as systolic blood pressure (SBP), diastolic blood pressure (DBP), serum triglyceride, serum high-density lipoprotein, and serum low-density lipoprotein. The LightGBM model achieved the lowest least mean square error (LMSE) for all indicators.

From the above review, we observed that Ethiopian data have never been explored before in diagnosing diabetes using artificial intelligence (AI) technology. Hence, an important goal of this project is to prepare a diabetes dataset for the application of machine-learning-based diabetes diagnosis serving two purposes: (*a*.) decision support for physicians and handling of potential diabetes conditions onset and (*b*.) improvement of DM detection coverage in countries with low physician density. From the existing work, we observed LightGBM and XGBoost ensemble classifiers are the most promising models for diabetes detection and even for diagnosing other diseases. However, XGBoost has a lower speed compared with LightGBM. The LightGBM algorithm features lower memory usage, higher speed and efficiency, compatibility with large datasets over XGBoost, and better accuracy than any other boosting algorithm [19]. LightGBM is almost seven times faster than XGBoost [19] and, hence, is a much better approach when working on large datasets. This makes LightGBM an interesting candidate for DM detection.

## 3. Materials and Methods

According to the WHO (World Health Organization) 2019 report [42], four gold standards of DM diagnostic tests are recommended, which are fasting plasma glucose (fasting blood sugar, FBS), 2-h post-load plasma glucose after a 75g oral glucose tolerance test (OGTT), HbA1c, and random blood glucose. Individuals with FBS values of ≥7.0mmol/L (126mg/dL), OGTT ≥11.1mmol/L (200mg/dL), HbA1c ≥6.5% (48mmol/mol), or a random blood glucose ≥11.1mmol/L (200mg/dL) are considered to have diabetes. In the case of Ethiopian hospitals, the majority of these diabetes diagnostics standards are practiced. Additionally, age, gender, body mass index (BMI), measured insulin, total cholesterol, the systolic value of blood pressure, the diastolic value of blood pressure, and low-density lipoprotein (LDL) cholesterol may be taken into account as optional attributes.

To achieve our goal, the study approach consists of five stages, which are (*i*.) overview of proposed approach, (*ii*.) diabetes data collection with the relevant attributes of the patients, (*iii*.) data preprocessing (cleaning), (*iv*.) evaluation metrics, and (*v*.) comparison of the proposed method with various machine learning classification techniques. Subsequently, we briefly discuss these procedures.

### 3.1. Overview of Proposed Approach

The proposed approach workflow includes the following steps:

1. Problem statement: Identify and solve scientific challenges to diagnose diabetes by machine learning in order to prevent or reduce its impact on physical and social well-being.
2. Relevant data collection: diabetes-related data were collected from Zewditu Memorial Hospital.
3. Diabetes dataset: the collected diabetes data were converted to machine learning model recognizable (tabular) format.
4. Data preprocessing: patterns underlying the data were visualized by box-plot and correlation heat-map. Irrelevant data elements and column values were removed and replaced, respectively. The correlation coefficient of each input variable (attributes) to the dependent variable (diabetes or not) was calculated to identify the important features. Each input variable has values in a different range; fast blood sugar (FBS) has minimum 60 and maximum 200 values; whereas, gender has binary values (minimum 0 and maximum 1) but machine learning algorithms recognize patterns numerically, meaning they give higher priority to attributes with large numerical values. By this scenario, FBS has higher priority over gender, which is logically not always true. To avoid such confusion, the attribute values were normalized in a common range using the Min-Max normalization technique [43]. Finally, the preprocessed dataset was split into training and test data samples.
5. Light Gradient Boosting Machine (LightGBM): the state-of-the-art LightGBM algorithm has been proposed to predict diabetes mellitus. Here, the LightGBM was optimized by calculating the optimal values of the hyperparameters using 10-fold cross-validation. Finally, we developed other classifier models viz. KNN, SVM, NB, Bagging (constructed on decision tree), RF, and XGBoost and compared the results with the optimal LightGBM model.

**Figure 1:**
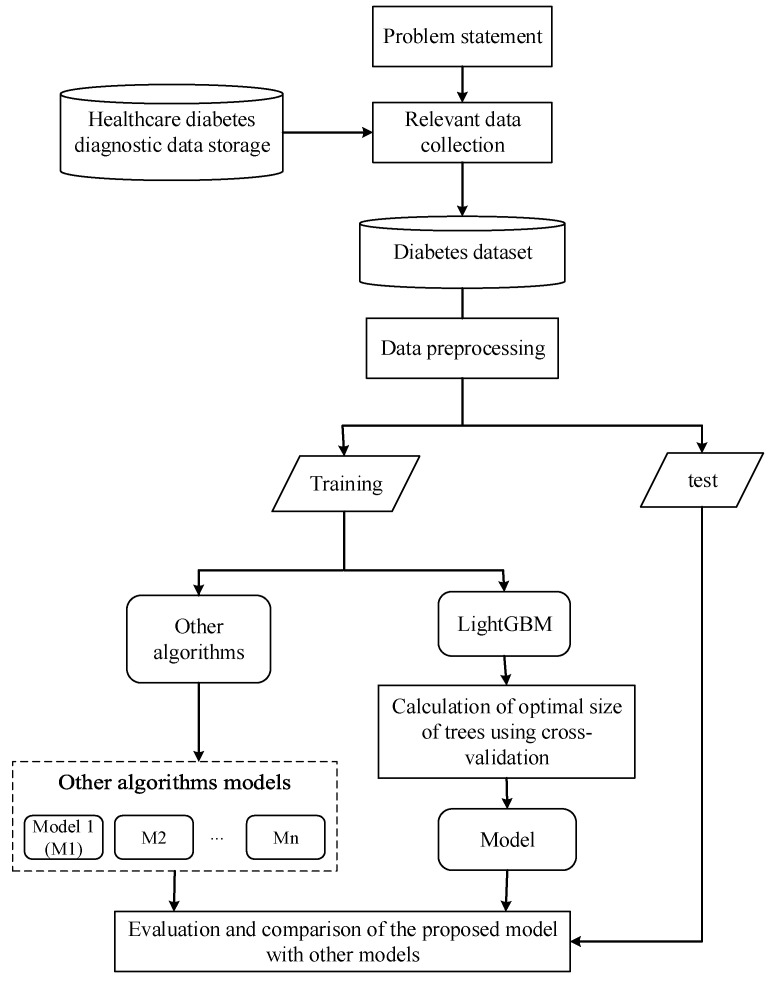
General framework of the proposed approach.

The general framework of the proposed approach is summarized in Figure 1.

#### LightGBM

Gradient Boosting Decision Tree (GBDT) is a common machine learning algorithm, which has effective implementations such as XGBoost and parallel Gradient Boosted Regression Trees pGBRT [44,45]. Although many engineering optimizations have been adopted in these implementations, for high-dimensional feature spaces and large data sizes, these implementations have comparably low efficiency and scalability. A major reason is that for each feature, they need to test all the data records to estimate the information gain of all possible split points, which requires very high computational time. Thus, to address these problems, Ke et al. [19] proposed LightGBM.

LightGBM is a gradient boosting framework that uses tree-based learning algorithms. It is designed to be distributed and efficient using two novel techniques: Gradient-based One-Side Sampling (GOSS) and Exclusive Feature Bundling (EFB) [19]. GOSS excludes a significant proportion of data instances with small gradients, and only uses the rest to estimate the information gain. Since the data records with larger gradients play a vital role in the computation of information gain, GOSS can obtain quite an accurate estimation of the information gain with a much smaller dataset. EFB is used for bundling mutually exclusive features to reduce the number of features. LightGBM is prefixed as Light because of its high speed. Compared to other existing Gradient Boosting Decision Tree algorithms, LightGBM has the advantages of faster training speed, higher efficiency, lower memory usage, better accuracy, being capability for handling large-scale data, and the support of parallel and GPU learning. LightGBM is a fast, distributed, high-performance gradient boosting framework based on a decision tree algorithm. It is used for ranking, classification, and many other machine learning tasks.

**Figure 2:**
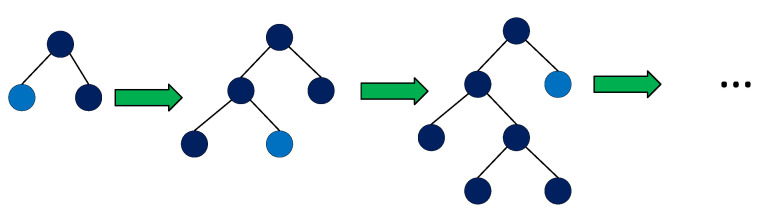
Leaf-wise tree growth in LightGBM [19].

**Figure 3:**
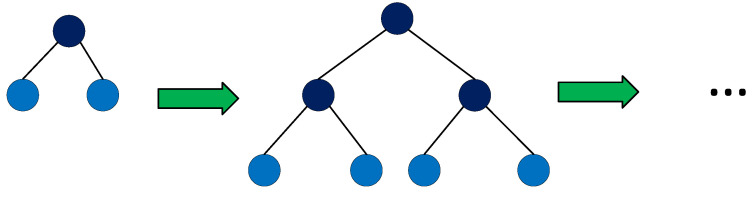
Level-wise tree growth in other boosting algorithms (such as in XGBoost) [19].

One of the characteristics that makes the LightGBM algorithm differ from other tree boosting algorithms is to split the tree leafwise as shown in Figure 2 with the best fit, whereas other boosting algorithms split the tree depthwise or levelwise, see Figure 3, rather than leafwise. So, when growing on the same leaf in LightGBM, the leafwise algorithm can reduce much more loss than the levelwise algorithm and, henceforth, results in much better accuracy, which is not met by any of the other boosting algorithms.

For a small size of data, leafwise growing may lead to an increase in complexity and result in overfitting [46]. To overcome this problem, we optimized the LightGBM algorithm for our medium size data ZMHDD (2000+) by precalculating the optimal values of the model’s hyperparameters to control the complexity of the LightGBM model. These are (*i*.) the number of iterations, (*ii*.) the maximum depth of the trees, and (*iii*.) the number of leaves. Hence, we retrieve the optimum number of trees, maximum depth of trees, and number of tree leaves. Details on the optimization process are given in Section 4.

### 3.2. Data Collection and Feature Selection

As of existing work studied in Section 2, Ethiopian data have never been explored before in machine-learning-based diabetes diagnosis. The physical examination data of 1030 people with DM and 1079 nondiabetic people were collected from Zewditu Memorial Hospital (administered under the city government of the Addis Ababa Health Bureau), and we called it the Zewditu Memorial Hospital diabetes dataset (ZMHDD). The relevance of the data was approved by the Ethical Clearance Committee of the city government of the Addis Ababa Health Bureau. The collected data contain about 23 indicators; however, many of these physical examination indicators had a weak correlation with DM. Researchers use different diabetes indicators in different contexts, as the diabetes condition depends on a societies’ food culture [18,30,31]. Hence, we consulted Ethiopian diabetologists about the diabetes condition and indicators in order to prepare our diabetes dataset ZMHDD. Since most of the collected diabetic data records are type 2 diabetes, we dropped the investigation of type 1 and Gestational data records, focusing on type 2 diabetes in this study. ZMHDD specifications are shown in Table 2 and selected indicators are shown in Table 1. From the available indicators, we selected those being significantly relevant to DM from the view of medicine and data correlation to the target variable (diabetic or not). We have considered invasive diabetes indicators such as insulin and fast blood sugar/glucose because they are commonly checked and accessible in Ethiopia. A future step for simplification may be to focus on noninvasive indicators. This may be desirable for regions without laboratory facilities and for broader risk assessments. The age of diabetic patients ranged from 12 to 90 years, and that of nondiabetic probands from 0.3 to 90 years.

### 3.3. Data Preprocessing

Although we have collected the diabetes physical examination dataset (ZMHDD) carefully, several instances were missing one or two feature values. In most data analysis studies, it is obvious to replace missing values by the mean of the corresponding feature (e.g., the column mean value for tabular data). However, for small datasets, replacing these missing values with the median of the corresponding features is better than replacing by mean [18]. Thus, the median of the corresponding feature was used to fill the missing feature values, which is a basic strategy. The median of diabetic and nondiabetic patients was computed separately to render the replaced values more representative.

The range of the feature values lay on different intervals, which can affect the results when building the machine learning model. Hence, the feature values are normalized using the Min-Max Normalization technique [43] to bound the values of all features between 0 and 1. Here, a given original feature set $X=\{x_{0},x_{1},\ldots ,x_{n-1}\}$ with *n* entries is normalized by

(1) $$x_{i}^{\prime }=\frac{x_{i}-\min\left(X\right)}{\max(X)-\min(X)}$$

where i∈{0,1,2,…,n−1}, min(X) is the minimum element of *X*, max(X) is its maximum element, and $X^{\prime }=\left\{x_{0}^{\prime },x_{1}^{\prime },\ldots ,x_{n-1}^{\prime }\right\}$ is the normalized feature set with $0\leq x_{i}^{\prime }\leq 1$.

### 3.4. Evaluation

To measure how well our model performs, different standard performance evaluation metrics [47]—i.e., accuracy, sensitivity, specificity, and the area under the receiver operating characteristic (AUC) curve—have been used. We also used the *k*-fold cross-validation method to split the dataset into *k* data subsets, with k−1 data subsets used as training sets and one of the subsets as the test set for one round of training. This allows for *k* constellations of model training and testing. Taking the average performance of the of the *k* training runs gives an indication of the generalization capability of the model on unknown data.

**Figure 4:**
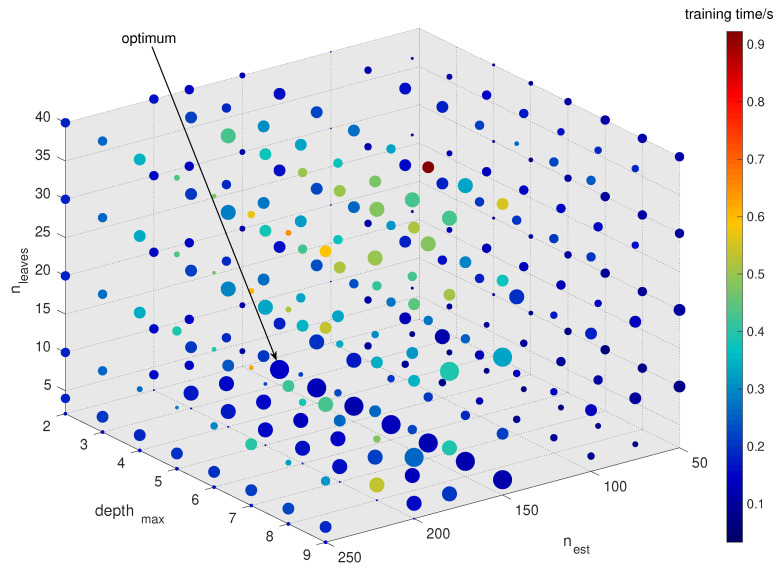
3D visualization of grid search result over the investigated space of hyperparameters $n_{est}$, $depth_{max}$ and $n_{leaves}$. Bubble sizes implicate the validation score (the larger, the better); the color indicates the required training time (the less, the better). The optimum configuration of a LightGBM model is at $n_{est}=150$, $depth_{max}=3$, and $n_{leaves}=4$ at a test accuracy of 0.9815 and a training time of 0.624 s}.

Specifically, the performance of the proposed model is evaluated on ZMHDD in two phases. First, 10-fold cross-validation is applied to each grid-search point in a grid search over three hyperparameters, as described in Section 4. This results in an optimum hyperparameter set of the LightGBM algorithm, as per Figure 4, and hence, determines the optimum model architecture. Since, due to cross-validation, the training of this architecture is based on a smaller dataset, its parametrization can be further aligned to data statistics by a training using the entire ZMHDD dataset separated into 80% training and 20% test data samples. Results will be discussed in the following section.

## 4. Experimental Results and Discussion

### 4.1. Experimental Results

The experimental analysis is carried out on the newly collected ZMHDD dataset. Using 10-fold cross-validation, the mean performance of a given model is evaluated. By variation over the hyperparameters, as listed in Section 3.1, the model architecture is optimized with regard to performance following the five steps outlined below.

**Figure 5:**
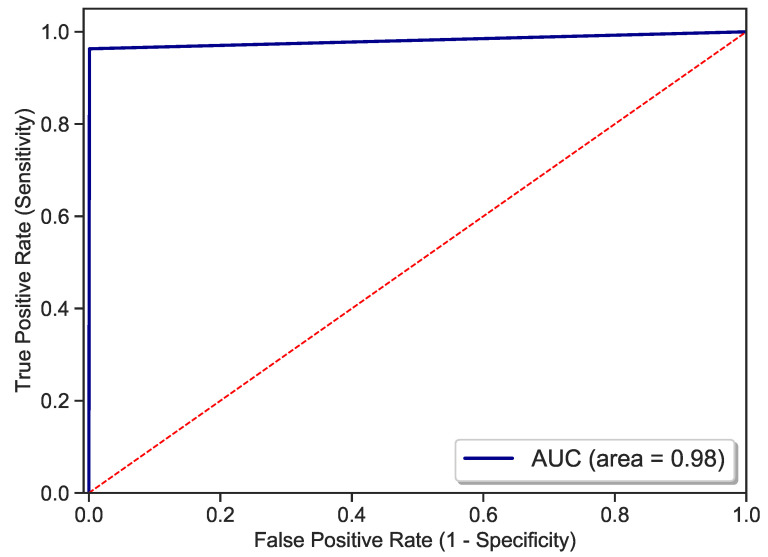
ROC curve.

1. Number of trees: The number of boosted trees or estimators will influence the LightGBM performance. To decide on the optimal number $n_{est}$ in case of the ZMHDD dataset, models with varying numbers of trees were constructed and evaluated.
2. Maximum tree depth: To avoid the occurrence of overfitting, we have to limit the maximum depth $depth_{max}$ of trees for tree-based models. This is especially important for small- or mid-sized datasets.
3. The number of tree leaves: is the main parameter to control the complexity of the tree model. Theoretically, we can set $n_{leaves}=2^{depth_{max}}$ to obtain the same number of leaves as a depth-wise tree. However, this is not always true in practice. Because a leaf-wise tree is typically much deeper than a depthwise tree for a fixed number of leaves. Unconstrained depth can induce overfitting [46]. Thus, when trying to optimize the num_leaves, we should let it be smaller than $2^{depth_{max}}$.
4. LightGBM model optimization: Several LightGBM models at variation of the $n_{est}$, $depth_{max}$, and $n_{leaves}$ parameters were constructed using 10-fold cross-validation grid search to define the optimal parametrization in the sense of a validation metric. Following the grid search, our model achieved the best accuracy of 98.15% at the configuration $n_{est}=150$, $depth_{max}=3$, and $n_{leaves}=4$. The 3D visualization of the 10-fold cross-validation grid search result is shown in Figure 4. The size of the bullets in Figure 4 indicates the validation score, the bubble colors indicate the training time.
5. Performance evaluation: Lastly, the performance of the designed LightGBM model is evaluated on the test data (20% of ZMHDD) using a training and test data splitting method [48]. Key metrics are given in Table 3 and Figure 5.

### 4.2. Comparison

To verify the effectiveness of LightGBM for the classification of diabetes mellitus, LightGBM’s performance is compared with additional six machine learning algorithms, namely, KNN, SVM, NB, Bagging (constructed on decision tree), RF, and XGBoost, applying the same database (ZMHDD). We computed the accuracy of these methods on the ZMHDD database. The comparison results are shown in Table 4.

Among these six additional methods, RF and XGBoost resulted a better accuracy of 96.9 and 96.5%, respectively. Conversely, the KNN method resulted in the lowest-in-class accuracy of 78.4% compared with the others. The optimized LightGBM model outperforms all other methods at 98.1% in terms of test accuracy.

To further evaluate the performance of the LightGBM model on our dataset ZMHDD, Table 5 compares the alternative methods with regard to accuracy, AUC, sensitivity, and specificity. With values of 98.1%, 98.1%, 99.9%, and 96.3%, LightGBM turns out to be best-in-class in all categories. Except for the significantly underperforming KNN, SVM, and NB models, the computational complexity for training is smallest among the better performing models. For testing, LightGBM has the fastest computation time among all models. This indicates that—as expected—the LightGBM tree model is overall simpler compared with the other ensemble learning methods. The effectiveness of the LightGBM-based approach comes from the fact that Light gradient boosting classifiers are a combined method of classifiers that can take advantage of the complementary manner of individual classifiers to improve performance. From the obtained results, we can say that LightGBM constitutes an important technique for the classification of medical data and, in particular, for the diagnosis of diabetic patients.

### 4.3. Limitations

A basic limitation of this study is imposed by restrictions in the availability of indicators considered. Levels of 2-h postload plasma glucose after a 75g oral glucose tolerance test (OGTT) and HbA1c test is one of the recommended indicators of diabetes. However, during data collection, we are unable to get enough OGTT and HbA1c test data due to the expensiveness of these tests, which constrains the accuracy of this study. Our study also considered some invasive diabetes indicators such as insulin and fast blood glucose, which may limit its application to self-testing in comparison with clinical use. Finally, we only have Ethiopia as a proxy for ethnicity. All of this affects our model’s detection capacity and generalization capability.

## 5. Conclusions

In this study, Ethiopian medical data (ZMHDD dataset) have been explored for the first time in the machine-learning-based diagnosis of diabetes mellitus. We have considered the early detection of diabetes mellitus by taking into account the significant risk factors related to DM. Mining knowledge from actual healthcare data can be useful to predict diabetic patients. Correlation coefficient and other data analysis techniques were used for feature selection. To detect DM effectively, we were interested in the development of a light gradient boosting machine (LightGBM) model for the classification of diabetic patients. Several LightGBM models were constructed, by varying the number and complexity of trees in the ensemble model, and evaluated according to their average accuracy by 10-fold cross-validation. Hence, the optimal LightGBM model could be determined. Finally, this model was compared to 6 reference models—KNN, SVM, NB, Bagging, RF, and XGBoost—in terms of accuracy, sensitivity, and specificity. The experimental results show that LightGBM outperforms these techniques for screening diabetes mellitus in all aspects. Therefore, the developed LightGBM model is deemed to be very effective to support physicians in the diagnosis of diabetes.

For the future, we aim to apply the proposed assistance system to real-time diabetes diagnosis systems. The proposed LightGBM model can be applied to other medical datasets to further validate the effectiveness and generalization capabilities of the model. In addition, it is better to place more emphasis on noninvasive diabetes indicators to detect diabetes in the general population.

## Figures and Tables

**Table 1:** Description of ZMHDD Attributes.

Diabetes Indicators (Attribute)	Description	Unit	Correlation to Class Variable	
Age	patient age	years	−0.03	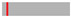
Gender	patient gender	male/female	0.014	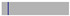
Insulin	hormone made by the pancreas organ that allows human body to use sugar (glucose) from food carbohydrates for energy conversion or storage	pmol/L	0.009	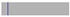
Systolic_BP	systolic value of blood pressure: indicates highest pressure exerted as blood pushes through heart	mmHg	0.16	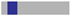
Diastolic_BP	diastolic value of blood pressure: indicates the pressure maintained by the arteries when the vessels are relaxed between heartbeats	mmHg	−0.045	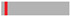
BMI	body mass index: a person’s weight in kilograms divided by the square of height in meters	kg/$m^{2}$	0.29	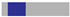
Total_Cholesterol	Total blood cholesterol: accumulated figure of all different blood fats (includes high-density lipoprotein (HDL), low-density lipoprotein (LDL) and 20% of the total triglycerides)	mg/dL	0.34	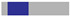
Low_Density_Lipoprotein	low-density lipoprotein (LDL) cholesterol: often known as ‘bad cholesterol’, because it can build up in blood vessels	mg/dL	0.11	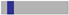
Pulse_Rate	a measurement of the heart rate, or the number of times the heart beats per minute; it also can indicate the heart rhythm and strength of the pulse.	bpm	0.19	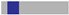
FBS	fasting blood sugar: blood sugar when a patient has not eaten or consumed any calories in the past 8 h (usually, this is done overnight)	mg/dL	0.37	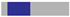
Class	observed diabetes status (0: nondiabetic, 1: diabetic)	–	1.00	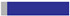

**Table 2:** Parameters of the ZMHDD diabetes datasets.

Dataset	#Instances	#Indicators	#Classes
ZMHDD	2109	10	2

**Table 3:** Key metrics of the optimized LightGBM model.

Metric	Value	
Accuracy	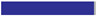	0.98
Sensitivity	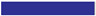	0.99
Specificity	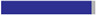	0.96

**Table 4:** Key metrics of the optimized LightGBM model.

Model	Accuracy	
KNN	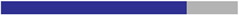	0.784
SVM	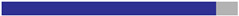	0.908
NB	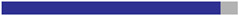	0.927
BG	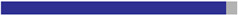	0.953
RF	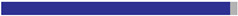	0.969
XGBoost	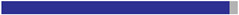	0.965
LightGBM	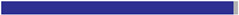	0.981

**Table 5:** Summary of experimental results and computational time on ZMHDD.

Algorithms	Accuracy	AUC	Sensitivity	Specificity	Computation Tims/s	
					Training	Testing
KNN	78.4%	77.8%	62.1%	93.6%	0.012	0.32
SVM	90.8%	90.4%	82.3%	98.6%	0.081	0.007
NB	92.7%	92.4%	85.2%	99.5%	0.05	0.006
BG	95.3%	95.2%	94.1%	96.3%	1.3	0.02
RF	96.9%	96.8%	93.6%	100%	2.5	0.011
XGBoost	96.4%	96.3%	92.6%	100%	2	0.004
LightGBM	98.1%	98.1%	99.9%	96.3%	0.624	0.0015

## Data Availability

Not applicable.
